# Burst ratio for a versatile traffic model

**DOI:** 10.1371/journal.pone.0272263

**Published:** 2022-08-01

**Authors:** Andrzej Chydzinski

**Affiliations:** Department of Computer Networks and Systems, Silesian University of Technology, Gliwice, Poland; Federal University of Pernambuco: Universidade Federal de Pernambuco, BRAZIL

## Abstract

We deal with a finite-buffer queue, in which arriving jobs are subject to loss due to buffer overflows. The burst ratio parameter, which reflects the tendency of losses to form long series, is studied in detail. Perhaps the most versatile model of the arrival stream is used, i.e. the batch Markovian arrival process (BMAP). Among other things, it enables modeling the interarrival time density function, the interarrival time autocorrelation function and batch arrivals. The main contribution in an exact formula for the burst ratio in a queue with BMAP arrivals and arbitrary service time distribution. The formula is presented in an explicite, ready-to-use form. Additionally, the impact of various system parameters on the burst ratio is demonstrated in numerical examples. The primary application area of the results is computer networking, where the complex nature of traffic has a deep impact on the burst ratio. However, due to the versatile arrival model, the results can be applied in other fields as well.

## 1 Introduction

When describing the loss process in a queue with finite buffer, two characteristics are especially useful: the loss ratio and the burst ratio. Each of them is easy to define and interpret, but when combined together, they present a quite comprehensive characterization of the loss process.

The loss ratio, *L*, is simply the long-run fraction of lost jobs. Equivalently, it is called the loss probability, or the blocking probability. It has been studied for a long time using mathematical, simulation and experimental approaches (references will be given in the next section).

The burst ratio, *B*, is a much newer characteristic, proposed in 2005, [1]. It is defined as the ratio of the average length of the series of losses in the stream of interest, to the hypothetical average length of series of losses in a process with every loss independent and of the same probability. Thus denoting the actual average length of the series of losses by $\bar{G}$, while the hypothetical average length by $\bar{K}$, we have:

$$\begin{array}{c} B=\bar{G}/\bar{K}. \end{array}$$ (1)

The denominator in this definition is used to extract the additional clustering of losses, caused by their mutual dependencies, from clustering caused by their bare random occurrence. Obviously, we can always have some series of losses formed randomly, without any additional reason.

It is easily seen that in a hypothetical loss process in which every loss is independent and of probability *L*, the series of consecutive losses has the average length of 1/(1 − *L*). Therefore, (1) can be rewritten as:

$$\begin{array}{c} B=\bar{G}\left(1-L\right). \end{array}$$ (2)

From the presented definition, it follows a simple interpretation of *B*. When *B* > 1, the losses have a tendency to cluster together in series. The larger *B*, the stronger this tendency is. When *B* < 1, the losses have a tendency to avoid each other and occur separately. When *B* = 1, the series of losses occur only by accidental occurrence of one loss next to the other. In applications, *B* = 2 can be considered hight, while *B* = 5 extremely high. The latter means that the average series of losses is 5 times longer than expected for independent, random losses, what must be caused by a very strong, positive correlation between the losses.

High values or *L* and *B* are usually not welcome and have a negative impact on the performance of a queueing system. It is interesting that in some applications the negative impact of *B* can be as important as the negative impact of *L*. For instance, formula (7-29) in [2] presents the combined negative impact of *L* and *B* on the packetized voice transmission (e.g. in the Internet).

Now, when analyzing the performance of a queueing system, it is crucial to model all its important features, including the interarrival time distribution, the interarrival time autocorrelation function (if non-zero), batch arrivals (if present) and the service time distribution. All these features are well known to have a deep impact on classic performance characteristics of the system, including its average queue length, response time and loss ratio. It is natural to suspect all these features to have a deep impact on the burst ratio as well, especially when combined together.

To the best of the author’s knowledge, there are no published studies, in which the burst ratio is analyzed in a queueing model having all the aforementioned features. The previous analytical studies of the burst ratio incorporated some of these features separately, but never combined together in one, complex model.

The main contribution of this paper is derivation of an explicite, ready-to-use formula for the burst ratio, in a model that enables mimicking all the aforementioned features of the real system. Namely, the burst ratio is derived for the queue with the batch Markovian arrival process, arbitrary service time distribution and arbitrary buffer size. In addition to theoretical results, the impact of the mentioned features of the system on its performance is demonstrated via numerical examples, based on five BMAP parameterizations of growing complexity.

The batch Markovian arrival process, BMAP, was initially called by its inventor, Marcel Neuts, a versatile point process [3]. This was done for good reasons. It is arguably the most versatile model of the point process, which remains analytically tractable. Firstly, by a proper parameterization, BMAP can be fitted as closely as needed to an arbitrary shape of the interarrival time distribution. Secondly, the autocorrelation function of the interarrival times can be fitted. Thirdly, the batch arrival structure can be modeled with arbitrary batch size distribution. Moreover, all these features can be modeled at the same time, using one BMAP parameterization. What is important, there are several known procedures for fitting BMAP parameters to the mentioned features of the arrival process (the literature will be recalled in the next section).

The primary field of application of the results presented herein is computer networking. Both BMAP and the burst ratio are well-known in networking, but so far have never been combined together, in one model. BMAP is very useful when modeling streams of packets in networks at least for two reasons. Firstly, it has been known for thirty years now, that the packet interarrival times can be strongly, positively autocorrelated, [4]. Secondly, the design of the commonly used Transmission Control Protocol (TCP) is based on the so called transmission window mechanism. In this mechanism, the packets are injected into the network rather in batches, than separately, [5]. BMAP can mimic these two important features of the traffic, autocorrelated arrivals and batches, at the same time. On the other hand, the burst ratio is especially important in real-time, multimedia transmissions, where losing several packets in a row degrades the quality of transmission perceived by a human user.

The paper is organized in the following manner. In Section 2, related work is recalled and characterized briefly. In Section 3, the queueing model is presented. In particular, a definition of the BMAP process is recalled, the operation of the queue is described, and a useful notation is introduced. Then, in Section 4, the main result of the paper is presented and proven, i.e. the formula for the burst ratio in a finite-buffer BMAP queue. A discussion of the numerical applicability of the main result follows then in Section 5. In Section 6, numerical examples are presented and commented. They include five BMAP parameterizations with different features and complexity, three different service time distributions, various buffer sizes and system loads. At the end of Section 6, a comparison of selected numerical results with those obtained in simulations is carried out. Finally, conclusions and suggestions of future work are gathered in Section 7.

## 2 Related work

To the best of the author’s knowledge, there are no published studies on the burst ratio in a finite-buffer queue fed by BMAP, or any other process able to mimic arbitrary interaarrival time distribution, batch arrivals and autocorrelation between interarrival times.

So far, the loss process in a finite-buffer BMAP queue has been studied with regard to the loss ratio only. Namely, an approximate analysis of the loss ratio in such model was carried out in [6]. The exact solution can be found in [7], both in transient and stationary regime. The loss ratio in a BMAP queue with special acceptance policies was computed in [8], while in [9] it was derived for a more general loss mechanism, based on the dropping function. Basic characteristics of the finite-buffer queue with BMAP arrivals, including the stationary distribution of the queue length and waiting time, can be found in [10, 11]. Finally, there are several papers, e.g. [12–14], making BMAPs useful in practice, by proposing methods for fitting BMAP parameters to the interarrival time distribution, autocorrelation function and the batch structure observed in a real process.

The burst ratio was introduced in [1]. Then it was analyzed in [15, 16] using an abstract model of losses, based on a two-state Markov chain. In such approach, the actual mechanism of losses is not modeled, what may have some deficiencies, [17]. Analytical studies of the burst ratio in a queueing model in which the losses are actually modeled by buffer overflows, can be found in [18–20], for different arrival processes. However, each of these processes has a far less rich structure and modeling capabilities than BMAP.

The burst ratio, although the simplest and most intuitive, is not the only possible characteristic describing the structure of losses. For instance, before the burst ratio was invented, the probability of losing *m* jobs among every *n* arrivals was analyzed in [21]. On the other hand, in [22] the distribution of the length of the first sequence of losses under a special acceptance discipline was analyzed.

All the articles cited so far were analytical studies, with mathematical modeling and queueing theory used as primary tools. It is worth mentioning, however, that the loss and burst ratio were also measured experimentally in telecommunication networks, [23–28].

The queueing discipline assumed in this paper enables partial admission of an arriving batch, i.e. if upon arrival of a batch, the buffer can accept only a part of the batch, this part is placed in the buffer, while the remaining part is rejected and lost. Such a discipline can be adopted, when each arrival within a batch constitutes a separate entity, e.g. a packet in networking, as discussed in the previous section. It is worth mentioning, however, that two other disciplines are studied in the literature: complete rejection and complete admission, see e.g. [8]. They are more difficult to solve, but also very practically motivated, e.g. by systems in which the whole batch constitutes an indivisible entity.

## 3 Queueing model

The BMAP process was proposed in [3]. The original, complicated definition was then rewritten in a simplified, but equivalent, form in [29]. This form is currently commonly used. Namely, BMAP is defined as Markov process (*N*(*t*), *J*(*t*)), *t* ≥ 0, where component *N*(*t*) ∈ {0, 1, …} represents the total number of job arrivals in (0, *t*), while component *J*(*t*) ∈ {1, …, *m*} is the state (phase) of the modulating process, a continuous-time Markov chain. The infinitesimal generator of process (*N*(*t*), *J*(*t*)) has the following form:

$$\begin{array}{c} {}[\begin{array}{ccccc} D_{0} & D_{1} & D_{2} & D_{3} & \cdot \;\cdot \\ & D_{0} & D_{1} & D_{2} & \cdot \;\cdot \\ & & D_{0} & D_{1} & \cdot \;\cdot \\ & & & \cdot & \cdot \;\cdot \end{array}], \end{array}$$

where *D*_*k*_, *k* ≥ 0 are *m* × *m* matrices such that every *D*_*k*_, *k* ≥ 1 is nonnegative, *D*_0_ has negative diagonal elements and nonnegative off-diagonal elements and $D=\sum_{k=0}^{\infty }D_{k}$ constitutes an irreducible infinitesimal generator, different than *D*_0_.

The counting function for the BMAP process is defined as:

$$\begin{array}{c} P_{i,j}\left(n,t\right)=P\left\{N\left(t\right)=n,J\left(t\right)=j|N\left(0\right)=0,J\left(0\right)=i\right\}, \end{array}$$ (3)

where P denotes probability.

If at some time *t* the BMAP process is in phase *i*, then the time after which there will be a change of the phase or an arrival of a batch is exponentially distributed with parameter λ_*i*_ = −(*D*_0_)_*ii*_. At this point, with probability *p*_*i*_(*j*, *k*) the phase will change to *j* or/and a batch of size *k* will arrive. It is easily verified that *p*_*i*_(0, *i*) = 0, $p_{i}\left(0,k\right)=\frac{1}{\lambda_{i}}{\left(D_{0}\right)}_{ik}$ for *k* ≠ *i*, and $p_{i}\left(j,k\right)=\frac{1}{\lambda_{i}}{\left(D_{j}\right)}_{ik}$ for *j* ≥ 1.

The rate of the BMAP process is equal to:

$$\begin{array}{c} \Lambda =\pi \sum_{k=1}^{\infty }kD_{k}e, \end{array}$$ (4)

where ***e*** = (1, …, 1)^*T*^ and ***π*** is the stationary vector for infinitesimal generator *D*, i.e. ***π**D* = **0**, ***πe*** = 1.

The queueing model of interest is the single-server queue with a finite buffer and BMAP arrivals, denoted as BMAP/G/1/*K* in Kendall’s notation. Namely, the arrival stream is BMAP, the buffer size is *K* (including service position), the distribution of the service time can assume any form, while its distribution function is denoted by *F*. The service discipline does not matter herein, so it can be FIFO, LIFO or any other. The finite-buffer assumption means that when there are *K* jobs present in the system (i.e. the buffer is full), a newly arriving job is rejected and lost. It cannot join the queue, leaves the system and never returns. If several jobs arrive simultaneously in a batch and there is a room in the buffer to accept only a part of the batch, this part is accepted and the remaining jobs are rejected.

The load of the queue is defined as:

$$\begin{array}{c} \rho =\Lambda \int_{0}^{\infty }tdF\left(t\right). \end{array}$$ (5)

The queue length at time *t*, including the service position if occupied, is denoted by *X*(*t*). As usually, it is assumed that the time origin corresponds to a service completion epoch. In the study, an important role will be played by the overflow period, by which we mean the continuous period of time time, when the buffer is full. It begins when the queue length reaches level *K*, making the buffer full, and ends, when free space in the buffer becomes available again.

Many derivations will be carried out in terms of *m*×*m* matrices and vectors of size *m*. To make the reading easier, we adopt the convention that matrices are denoted with capital letters, while vectors with lowercase letters. In particular, the following *m* × *m* matrices will be exploited:

$$\begin{array}{c} I=\text{the}\;\text{identity}\;\text{matrix},\\ 0=\text{the}\;\text{zero}\;\text{matrix},\\ 1=\text{the}\;\text{matrix}\;\text{of}\;1'\text{s}, \end{array}$$

$$\begin{array}{c} Y_{k}\left(s\right)={\left[\frac{\lambda_{i}p_{i}\left(k,j\right)}{s+\lambda_{i}}\right]}_{i,j},\;\;\;Y_{k}=Y_{k}\left(0\right), \end{array}$$ (6)

$$\begin{array}{c} A_{k}\left(s\right)={\left[\int_{0}^{\infty }e^{-st}P_{i,j}\left(k,t\right)dF\left(t\right)\right]}_{i,j},\;\;\;A_{k}=A_{k}\left(0\right), \end{array}$$ (7)

$$\begin{array}{c} E_{k}\left(s\right)={\left[\int_{0}^{\infty }e^{-st}P_{i,j}\left(k,t\right)\left(1-F\left(t\right)\right)dt\right]}_{i,j}, \end{array}$$ (8)

$$\begin{array}{c} {\bar{A}}_{n}\left(s\right)=\sum_{k=n}^{\infty }A_{k}\left(s\right)=A_{0}\left(s\right)-\sum_{k=0}^{n-1}A_{k}\left(s\right),\;\;\;{\bar{A}}_{k}={\bar{A}}_{k}\left(0\right), \end{array}$$ (9)

$$\begin{array}{c} B_{n}\left(s\right)=A_{n+1}\left(s\right)-{\bar{A}}_{n+1}\left(s\right){\left({\bar{A}}_{0}\left(s\right)\right)}^{-1}, \end{array}$$ (10)

$$\begin{array}{c} R_{0}\left(s\right)=0,\;\;\;R_{1}\left(s\right)=A_{0}^{-1}\left(s\right), \end{array}$$

$$\begin{array}{c} R_{k+1}\left(s\right)=A_{0}^{-1}\left(s\right)(R_{k}\left(s\right)-\sum_{i=0}^{k}A_{i+1}\left(s\right)R_{k-i}\left(s\right)),\;\;k\geq 1, \end{array}$$ (11)

$$\begin{array}{c} R_{k}=R_{k}\left(0\right), \end{array}$$ (12)

and the following column vectors of size *m*:

e=thevectorof1's,

$$\begin{array}{c} c_{k}\left(s\right)=\frac{1}{s}\sum_{i=K-k}^{\infty }\left(i-K+k\right)A_{i}\left(s\right)e+\sum_{i=K-k}^{\infty }\left(i-K+k\right)E_{i}\left(s\right)e. \end{array}$$ (13)

## 4 Burst ratio

**Theorem 1**. *In a queue with BMAP arrivals and a finite buffer of size K, the burst ratio is equal to*:

$$\begin{array}{c} B=\frac{1-L}{1-ur(0)}\sum_{l=1}^{\infty }lur\left(l\right), \end{array}$$ (14)

*where*

$$\begin{array}{cc} r(l)= & {\left(\sum_{k=0}^{K}R_{K-k}A_{k}-\sum_{k=1}^{K}\sum_{j=0}^{k}Y_{K-k}R_{k-j}A_{j}\right)}^{-1}\\ & \cdot \left(\sum_{k=1}^{K}R_{K-k}A_{k+l}-\sum_{k=1}^{K}\sum_{j=1}^{k}Y_{K-k}R_{k-j}A_{j+l}+\sum_{k=K}^{K+l}Y_{k}A_{K+l-k}\right)e, \end{array}$$ (15)

***u*** *is the unique solution of the system*:

$$\begin{array}{c} uV=u,\;\;\;ue=1, \end{array}$$ (16)

*where*

$$\begin{array}{cc} V= & {\left(\sum_{k=0}^{K}R_{K-k}A_{k}-\sum_{k=1}^{K}\sum_{j=0}^{k}Y_{K-k}R_{k-j}A_{j}\right)}^{-1}\\ & \cdot \left(\sum_{k=1}^{K}R_{K-k}{\bar{A}}_{k}-\sum_{k=1}^{K}\sum_{j=1}^{k}Y_{K-k}R_{k-j}{\bar{A}}_{j}+\sum_{k=K}^{\infty }Y_{k}{\bar{A}}_{0}\right), \end{array}$$ (17)

*and*

$$\begin{array}{c} L=\underset{s\to 0+}{\text{lim}}s^{2}Q^{-1}\left(s\right)q\left(s\right)\cdot \left(1,0\ldots ,0\right)/\Lambda , \end{array}$$ (18)

*with*

$$\begin{array}{cc} Q\left(s\right)= & R_{K+1}\left(s\right)A_{0}\left(s\right)+\sum_{k=0}^{K}R_{K-k}\left(s\right)B_{k}\left(s\right)-\sum_{k=0}^{K}Y_{K-k}\left(s\right)R_{k+1}\left(s\right)A_{0}\left(s\right)\\ & -\sum_{k=0}^{K}\sum_{l=0}^{k}Y_{K-k}\left(s\right)R_{k-l}\left(s\right)B_{l}\left(s\right)-\sum_{k=K+1}^{\infty }Y_{k}\left(s\right), \end{array}$$ (19)

$$\begin{array}{cc} q\left(s\right)= & \sum_{k=0}^{K}\sum_{l=0}^{k}Y_{K-k}\left(s\right)R_{k-l}\left(s\right)\left[{\bar{A}}_{l+1}\left(s\right){\left({\bar{A}}_{0}\left(s\right)\right)}^{-1}c_{K}\left(s\right)-c_{K-l}\left(s\right)\right]\\ & -\sum_{k=0}^{K}R_{K-k}\left(s\right)\left[{\bar{A}}_{k+1}\left(s\right){\left({\bar{A}}_{0}\left(s\right)\right)}^{-1}c_{K}\left(s\right)-c_{K-k}\left(s\right)\right]+\sum_{k=1}^{\infty }kY_{K+k}\left(s\right)e/s. \end{array}$$ (20)

Before the formal proof is given, it is worth presenting its general structure. The proof will be divided into three parts. In the first part, we will find the distribution of the modulating chain at the end of the first overflow period, depending on the initial queue length and the initial state of the modulating chain. This distribution will be then used to derive matrix *V*, which is the transition matrix of a discrete-time Markov chain embedded at ends of overflow periods. Having matrix *V*, we can easily obtain its stationary distribution, ***u***. In the second part of the proof, we will derive the distribution of the number of losses, if any, during the first overflow period, depending on the initial queue length and the initial state of the modulating chain. Having this distribution and vector ***u***, we will be able to find the distribution of the number of losses in an overflow period in the stationary regime. The third part of the proof will be short and easy. Using the results obtained in the previous two parts we will derive a formula for the average length of the series of losses, $\bar{G}$. Finally, combining (2) with known results on the loss ratio in the finite-buffer BMAP queue, we will complete the proof.

Proof of Theorem 1. We can assume without loss of generality, that initially the buffer is not full, i.e. *X*(0) < *K*. Let *t*_0_ = 0 and *t*_*k*_, *k* ≥ 1 denote the end of the *k*-th overflow period. Moreover, let *η*_*k*_ be the state of the modulating chain at the time *t*_*k*_:

$$\begin{array}{c} \eta_{k}=J\left(t_{k}\right),\;\;\;k\geq 0. \end{array}$$ (21)

Let us define matrix *V*_*n*_ as follows:

$$\begin{array}{c} V_{n}={\left[P\{\eta_{1}=j|X\left(0\right)=n,J\left(0\right)=i\}\right]}_{i,j},\;\;\;\;\;0\leq n<K,\;\;1\leq i,j\leq m. \end{array}$$ (22)

As we can see, each row of *V*_*n*_ contains the distribution of the modulating chain at the end of the first overflow period, depending on the initial state of the modulating chain. We will find the formula for *V*_*n*_ now.

If the system is initially non-empty, then we have for 0 < *n* < *K*, 1 ≤ *i* ≤ *m*:

$$\begin{array}{cc} P\{\eta_{1}=j| & X\left(0\right)=n,J\left(0\right)=i\}\\ & \;=\sum_{l=1}^{m}\sum_{k=0}^{K-n-1}\int_{0}^{\infty }P\left\{\eta_{1}=j|X\left(0\right)=n+k-1,J\left(0\right)=l\right\}P_{i,l}\left(k,u\right)dF\left(u\right)\\ & \;\;+\sum_{k=K-n}^{\infty }\int_{0}^{\infty }P_{i,j}\left(k,u\right)dF\left(u\right), \end{array}$$ (23)

where *P*_*i*, *l*_(*k*, *u*) is defined in (3). Eq (23) is obtained by conditioning on the first departure time, *u*. If by the time *u* there are no more than *K* − *n* − 1 new arrivals, then the buffer does not get full by the time *u* and the new queue length at time *u* is *n* + *k* − 1. This is expressed by the first part of (23), together with all possible changes of the modulating state from *i* at the beginning, to *l* at time *u*. If by the time *u* there are at least *K* − *n* new arrivals, then the buffer gets full by the time *u* and *u* is the end of the first overflow period, *u* = *t*_1_. Thus *η*_1_ is equal to the modulating state at the time *u*, what is covered by the second part of (23).

If the system is initially empty, then we obtain for 1 ≤ *i* ≤ *m*:

$$\begin{array}{cc} P\{\eta_{1}=j|X\left(0\right)=0,J\left(0\right)=i\}= & \sum_{l=1}^{m}\sum_{k=0}^{K-1}p_{i}\left(k,l\right)P\left\{\eta_{1}=j|X\left(0\right)=k,J\left(0\right)=l\right\}\\ & +\sum_{l=1}^{m}\sum_{k=K}^{\infty }p_{i}\left(k,l\right)\int_{0}^{\infty }P\left\{J\left(u\right)=j|J\left(0\right)=l\right\}dF\left(u\right), \end{array}$$ (24)

where *p*_*i*_(*k*, *l*) is defined in the third paragraph of Section 3. Eq (24) is obtained by conditioning on the size of the first arriving batch, *k*. If *k* ≤ *K* − 1, then the buffer does not get full upon the arrival of the first batch and the new queue length is *k*, what is covered by the first part of (24). If *k* ≥ *K*, then the buffer gets full upon the first arrival. At this moment, both the first service and the first overflow period begin. Thus the second part of (24) expresses the possible change of the modulating state during one service time, equal to the overflow period in this case. Obviously, we have:

$$\begin{array}{c} \int_{0}^{\infty }P\left\{J\left(u\right)=j|J\left(0\right)=i\right\}dF\left(u\right)=\sum_{q=1}^{\infty }\int_{0}^{\infty }P_{i,j}\left(q,t\right)dF\left(t\right). \end{array}$$ (25)

Therefore, (23), (24) and (25) yield:

$$\begin{array}{c} V_{n}=\sum_{k=0}^{K-n-1}A_{k}V_{n+k-1}+{\bar{A}}_{K-n},\;\;\;\;\;0<n<K, \end{array}$$ (26)

$$\begin{array}{c} V_{0}=\sum_{k=0}^{K-1}Y_{k}V_{k}+Z, \end{array}$$ (27)

where

$$\begin{array}{c} Z=\sum_{k=K}^{\infty }Y_{k}{\bar{A}}_{0}, \end{array}$$ (28)

and matrices *A*_*k*_, ${\bar{A}}_{k}$ and *Y*_*k*_ are defined in (6), (7) and (9), respectively. Using a new indexing, ${\bar{V}}_{n}=V_{K-n}$, yields:

$$\begin{array}{c} \sum_{k=-1}^{n-1}A_{k+1}{\bar{V}}_{n-k}-{\bar{V}}_{n}=H_{n},\;\;\;\;\;\;\;0<n<K, \end{array}$$ (29)

with

$$\begin{array}{c} H_{n}=A_{n}{\bar{V}}_{1}-{\bar{A}}_{n}, \end{array}$$ (30)

and

$$\begin{array}{c} {\bar{V}}_{K}=\sum_{k=1}^{K}Y_{K-k}{\bar{V}}_{k}+Z. \end{array}$$ (31)

From Lemma 3.2.1 of [30], it follows that the general solution of system (29) can be expressed as:

$$\begin{array}{c} {\bar{V}}_{n}=R_{n}C+\sum_{k=1}^{n}R_{n-k}H_{k},\;\;\;\;\;n\geq 1, \end{array}$$ (32)

where matrices *R*_*k*_ are defined in (11) while *C* is a constant matrix. Putting *n* = 1 to (32) leads to:

$$\begin{array}{c} C=A_{0}{\bar{V}}_{1}. \end{array}$$ (33)

From (33) we obtain then:

$$\begin{array}{c} {\bar{V}}_{n}=\sum_{k=0}^{n}R_{n-k}A_{k}{\bar{V}}_{1}-\sum_{k=1}^{n}R_{n-k}{\bar{A}}_{k}. \end{array}$$ (34)

Exploiting formula (34) for *n* = *K* and applying (31) yields:

$$\begin{array}{c} \sum_{k=0}^{K}R_{K-k}A_{k}{\bar{V}}_{1}-\sum_{k=1}^{K}R_{K-k}{\bar{A}}_{k}=\sum_{k=1}^{K}Y_{K-k}\left(\sum_{l=0}^{k}R_{k-l}A_{l}{\bar{V}}_{1}-\sum_{l=1}^{k}R_{k-l}{\bar{A}}_{l}\right)+Z, \end{array}$$ (35)

what makes it possible to derive ${\bar{V}}_{1}$:

$$\begin{array}{c} {\bar{V}}_{1}=V_{K-1}=\\ ={\left(\sum_{k=0}^{K}R_{K-k}A_{k}-\sum_{k=1}^{K}\sum_{l=0}^{k}Y_{K-k}R_{k-l}A_{l}\right)}^{\;-1}\;\left(\sum_{k=1}^{K}R_{K-k}{\bar{A}}_{k}-\;\sum_{k=1}^{K}\sum_{l=1}^{k}Y_{K-k}R_{k-l}{\bar{A}}_{l}+Z\right). \end{array}$$ (36)

Defining *V* = *V*_*K*−1_, we have just proven formula (17), which the same as (36). This completes the first part of the proof. Note that we can compute also *V*_*n*_ for arbitrary *n* using (34), but this will not be needed in the proof.

Now, it is easy to see that sequence *η*_*k*_ defined in (21) constitutes a discrete-time Markov chain. If the initial queue length is *n*, then the transition of this chain in the first step, *η*_0_ → *η*_1_, is governed by matrix *V*_*n*_, while transition in every next step is governed by matrix *V*. This is due to the fact that just after an overflow period, the queue length is always *K* − 1. Moreover, each end of the overflow period constitutes a regeneration point for the combined process of the queue length and the modulating chain. Hence, the queue length *K* − 1 is a new initial queue length for the next overflow period. Therefore, the stationary distribution of chain *η*_*k*_ must be ***u***, where ***u*** is a unique solution of system (16).

In the second part of the proof, we will derive the distribution of the number of losses during the first overflow period. Let *ζ*_*k*_, *k* ≥ 1 denote the number of losses during the *k*-th overflow period. If the queue is not empty at *t* = 0, we obtain for 0 < *n* < *K*, 1 ≤ *i* ≤ *m*:

$$\begin{array}{c} P\{\zeta_{1}=l|X\left(0\right)=n,J\left(0\right)=i\}\\ \;=\sum_{j=1}^{m}\sum_{k=0}^{K-n-1}\int_{0}^{\infty }P\left\{\zeta_{1}=l|X\left(0\right)=n+k-1,J\left(0\right)=j\right\}P_{i,j}\left(k,u\right)dF\left(u\right)\\ \;\;+\sum_{j=1}^{m}\int_{0}^{\infty }P_{i,j}\left(K-n+l,u\right)dF\left(u\right). \end{array}$$ (37)

Eq (37) is constructed in a similar way to (23), i.e. using the total probability formula with respect to the first departure time, *u*. Then, if by the time *u* there are no more than *K* − *n* − 1 arrivals, the new queue length at time *u* is *n* + *k* − 1. If by the time *u* there are more than *K* − *n* − 1 arrivals, then the buffer gets overflowed by the time *u*. In this case, in order to have *ζ*_1_ = *l*, the total number of arrivals by the time *u* must be *K* − *n* + *l*. In the case *X*(0) = 0, we have now:

$$\begin{array}{cc} P\{\zeta_{1}=l|X\left(0\right)=0,J\left(0\right)=i\}= & \sum_{j=1}^{m}\sum_{k=0}^{K-1}p_{i}\left(k,j\right)P\left\{\zeta_{1}=l|X\left(0\right)=k,J\left(0\right)=j\right\}\\ & +\sum_{j=1}^{m}\sum_{k=K}^{K+l}p_{i}\left(k,j\right)\int_{0}^{\infty }\sum_{a=1}^{m}P_{j,a}\left(K+l-k,u\right)dF\left(u\right), \end{array}$$ (38)

which is obtained in the same way as (24), i.e. conditioning on the size of the first arriving batch. Denoting:

$$\begin{array}{c} r_{n,i}\left(l\right)=P\left\{\zeta_{1}=l|X\left(0\right)=n,J\left(0\right)=i\right\},\;\;\;l=0,1,2,\ldots , \end{array}$$ (39)

$$\begin{array}{c} r_{n}\left(l\right)={\left[r_{n,1}\left(l\right),\ldots ,r_{n,m}\left(l\right)\right]}^{T}, \end{array}$$ (40)

from (37) and (38) we have:

$$\begin{array}{c} r_{n}\left(l\right)=\sum_{k=0}^{K-n-1}A_{k}r_{n+k-1}\left(l\right)+A_{K+l-n}e,\;\;\;\;\;0<n<K, \end{array}$$ (41)

$$\begin{array}{c} r_{0}\left(l\right)=\sum_{k=0}^{K-1}Y_{k}r_{k}\left(l\right)+\sum_{k=K}^{K+l}Y_{k}A_{K+l-k}e. \end{array}$$ (42)

It is easily seen, that the system (41) and (42) has the same form as system (26) and (27). Proceeding in the same way as we proceeded when solving (26) and (27), we will arrive at the following solution:

$$\begin{array}{c} r_{K-1}\left(l\right)=r\left(l\right), \end{array}$$ (43)

where ***r***(*l*) is given in (15). This completes the second part of the proof.

Now, knowing that at the end of an overflow period the queue length is *K* − 1, and that the stationary distribution of the state of the modulating chain is ***u***, we can conclude immediately that the distribution of the number of losses during an overflow period in the stationary regime is ***ur***(*l*). Note however, that the number of losses during an overflow period is not equivalent to the length of the series of losses. If the former is zero, which is possible, then the series does not occur at all. To compensate for this fact, we have to exclude such a case when computing the average length of the series of losses, $\bar{G}$. Hence, we obtain:

$$\begin{array}{c} \bar{G}=\frac{1}{1-ur\left(0\right)}\sum_{l=1}^{\infty }lur\left(l\right). \end{array}$$ (44)

Finally, to compute the burst ratio, we have to apply (2), which contains the loss ratio, *L*. The formula for the loss ratio in a finite-buffer BMAP queue has been derived in [7] and is recalled herein in (18)–(20). The general idea of the proof of (18) is the following. First, a system of integral equations for the average number of losses in interval (0, *t*] is constructed. Then, a solution of this system is obtained in terms of the Laplace transform. Finally, the long-term loss ratio is obtained using the limiting behaviour of the Laplace transform as *s* → 0+, which reflects the behaviour of the original characteristic as *t* → ∞. The details can be found in [7].

This completes the proof of Theorem 1.

## 5 Computational remarks

From the practical point of view, it is important that there are no significant difficulties when using Theorem 1 in numerical calculations. Although some of the formulas are long and complex, they are at the same time easy to program on a computer.

Note that matrices *A*_*k*_(*s*) and *E*_*k*_(*s*) can be computed by means of the well-known uniformization method, [29]. Applying this method we get:

$$\begin{array}{c} A_{k}\left(s\right)=\sum_{j=0}^{\infty }\gamma_{j}\left(s\right)K_{k,j},\;\;\;\gamma_{j}\left(s\right)=\int_{0}^{\infty }\frac{e^{-\left(\theta +s\right)t}{\left(\theta t\right)}^{j}}{j!}dF\left(t\right), \end{array}$$ (45)

$$\begin{array}{c} E_{k}\left(s\right)=\sum_{j=0}^{\infty }\delta_{j}\left(s\right)K_{k,j}\;\;\;\delta_{j}\left(s\right)=\int_{0}^{\infty }\frac{e^{-\left(\theta +s\right)t}{\left(\theta t\right)}^{j}}{j!}\left(1-F\left(t\right)\right)dt, \end{array}$$ (46)

where *θ* = max_*i*_{(−*D*_0_)_*ii*_} and matrices *K*_*n*,*j*_ are defined as follows:

$$\begin{array}{ccc} K_{0,0} & = & I,\;\;\;\;\;\;\;\;K_{n,0}=0,\;\;n\geq 1,\\ K_{0,j+1} & = & K_{0,j}\left(I+\theta^{-1}D_{0}\right),\\ K_{n,j+1} & = & \theta^{-1}\sum_{i=0}^{n-1}K_{i,j}D_{n-i}+K_{n,j}\left(I+\theta^{-1}D_{0}\right). \end{array}$$ (47)

Integrals *γ*_*j*_(*s*) and *δ*_*j*_(*s*) are well known in the queueing theory. They can be computed symbolically for several commonly used classes of distribution *F*. In the remaining cases, numerical integration can be used.

The limit in (18) can be obtained simply by using a small value of *s*, and checking if the result alters when *s* smaller by a few orders of magnitude is used, e.g. *s*/10, *s*/100, *s*/1000. In all the numerical results presented in the next section, the value *s* = 10^−8^ was used, what provided at least 5 correct decimal digits of *B*.

Note also that in practice we usually have a finite number of different sizes of batches in the system. If this is the case, all the series involving *Y*_*k*_(*s*) become finite, rather than infinite, in particular the last terms of (17), (19) and (20).

Finally, it is worth mentioning that the loss ratio present in Theorem 1 can be also calculated in a different way than by means of (18)–(20). Namely, using the results of [10], one can compute the stationary probability of an empty system, *p*_0_, and apply the well-known formula for *L*, i.e.:

$$\begin{array}{c} L=1-\frac{1-p_{0}}{\rho }. \end{array}$$ (48)

Such method, however, requires inversion of an *mK*×*mK* matrix when computing *p*_0_. Thus the method is of *O*(*m*^3^*K*^3^) computational complexity. As it is easy to check, the method based on formulas (18)–(20) does not involve any *mK* × *mK* matrices and is of significantly smaller complexity, *O*(*m*^3^*K*^2^).

## 6 Examples

In this section we use five BMAP parameterizations with *m* = 3 to demonstrate, how different features of the arrival stream can influence the burst ratio.

Namely, the following parameterizations are used:

BMAP_1_: *D*_0_ = −6.66666666 ⋅ *I*, *D*_1_ = 6.66666666 ⋅ *I*.
BMAP_2_:
  $$\begin{array}{c} D_{0}=\left[\begin{array}{ccc} -2.66407491 & 0.21318153 & 0.06876823\\ 0.28194977 & -4.12978130 & 0.28194977\\ 0.07564506 & 0.07564506 & -14.6406033 \end{array}\right], \end{array}$$
  $$\begin{array}{c} D_{1}=\left[\begin{array}{ccc} 1.30324098 & 0.60086202 & 0.47802213\\ 0.28641400 & 2.92428551 & 0.35518224\\ 0.80716673 & 0.28882659 & 13.3933198 \end{array}\right]. \end{array}$$
BMAP_3_: *D*_0_ = −*I*, *D*_2_ = 0.02222222 ⋅ **1**, *D*_4_ = 0.07777778 ⋅ **1**, *D*_8_ = 0.23333333 ⋅ **1**.
BMAP_4_:
  $$\begin{array}{c} D_{0}=\left[\begin{array}{ccc} -0.39961124 & 0.03197723 & 0.01031523\\ 0.04229246 & -0.61946720 & 0.04229246\\ 0.01134675 & 0.01134675 & -2.19609050 \end{array}\right], \end{array}$$
  $$\begin{array}{c} D_{2}=\left[\begin{array}{ccc} 0.14544482 & 0.01134675 & 0.02166199\\ 0.01134675 & 0.03197723 & 0.02166199\\ 0.02166199 & 0.03197723 & 0.05260770 \end{array}\right], \end{array}$$
  $$\begin{array}{c} D_{4}=\left[\begin{array}{ccc} 0.01134675 & 0.02166199 & 0.01134675\\ 0.01134675 & 0.33111906 & 0.01134675\\ 0.04229246 & 0.01134675 & 0.02166199 \end{array}\right], \end{array}$$
  $$\begin{array}{c} D_{8}=\left[\begin{array}{ccc} 0.03869456 & 0.05712054 & 0.03869456\\ 0.02026858 & 0.07554653 & 0.02026858\\ 0.05712054 & 0.00000000 & 1.93472829 \end{array}\right]. \end{array}$$
BMAP_5_:
  $$\begin{array}{c} D_{0}=\left[\begin{array}{ccc} -45.5935855 & 1.95261616 & 0.19526161\\ 0.01952616 & -4.55935855 & 0.19526161\\ 0.00195261 & 0.01952616 & -0.45593586 \end{array}\right], \end{array}$$
  $$\begin{array}{c} D_{2}=\left[\begin{array}{ccc} 0.06508720 & 0.52069762 & 5.20697622\\ 0.52069762 & 0.00065087 & 0.05792761\\ 0.05076801 & 0.00650872 & 0.00065087 \end{array}\right]\;, \end{array}$$
  $$\begin{array}{c} D_{4}=\left[\begin{array}{ccc} 0.06508720 & 0.52069762 & 5.20697622\\ 0.52069762 & 0.00065087 & 0.05792761\\ 0.05076801 & 0.00650872 & 0.00065087 \end{array}\right], \end{array}$$
  $$\begin{array}{c} D_{8}=\left[\begin{array}{ccc} 0.35797962 & 2.86383692 & 28.6383692\\ 2.86383692 & 0.00357979 & 0.31860186\\ 0.27922410 & 0.03579796 & 0.00357979 \end{array}\right]. \end{array}$$

**Fig 1:**
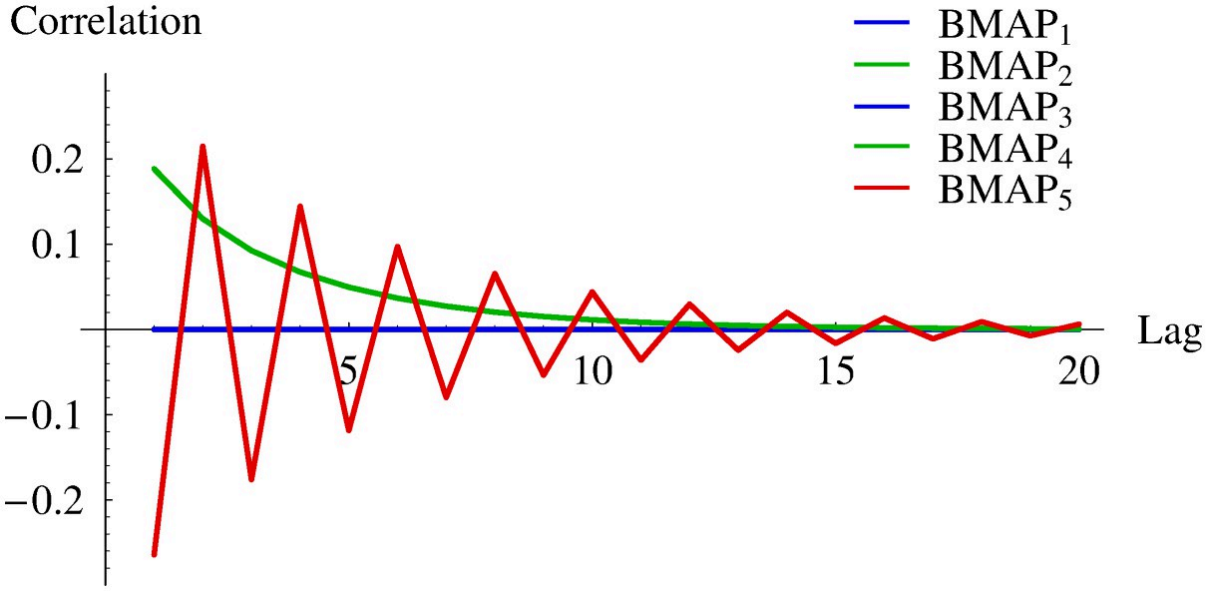
The autocorrelation function of interarrival times.

These BMAPs have growing complexity in the following sense. BMAP_1_ has neither batch arrivals, nor autocorrelated interarrival times. BMAP_2_ has no batch arrivals, but has positively autocorrelated interarrival times (see Fig 1 for its autocorrelation function). BMAP_3_ has batch arrivals, but no autocorrelation of interarrival times. BMAP_4_ has batch arrivals and positively autocorrelated interarrival times (see Fig 1). Finally, BMAP_5_ has batch arrivals and autocorrelated interarrival times, with the autocorrelation function having alternating signs (see Fig 1). To make the comparisons fair, all five BMAPs have the same arrival rate, *Λ* = 6.666666. In all the cases with batch arrivals, i.e. BMAP_3_-BMAP_5_, the same batch sizes are used, i.e. 2, 4 and 8, with the same batch arrival rate of 1 and the same average batch size of 6.666666. Finally, BMAP_2_ and BMAP_4_ share the same autocorrelation function (see Fig 1 again).

Four distributions of the service time are used. Namely, *F*_1_ is a constant service time equal to 1/6.666666, *F*_2_ is a uniform distribution on the interval (0, 2/6.666666), *F*_3_ is Erlang distribution with parameters (2, 13.333333), while *F*_4_ is a hyper-exponential distribution with parameters (*p*_1_, *p*_2_) = (0.95, 0.05), (λ_1_, λ_2_) = (9.5, 1). As we can see, each of these distributions has the average value of 1/6.666666, thus each one of them combined with each BMAP given above gives *ρ* = 1. Moreover, distributions *F*_1_-*F*_4_ have the following coefficients of variation: 0, 0.57, 0.71 and 2.09, respectively.

**Table 1:** The burst ratio for different BMAPs and service time distributions.

service	constant service	uniform service	Erlang service	hyper-exp. s̊ervice
BMAP_1_, *K* = 25	1.3317	1.5011	1.6377	4.3665
BMAP_2_, *K* = 25	1.6558	1.9525	2.0754	3.9805
BMAP_3_, *K* = 25	3.6778	3.8124	3.8802	5.3903
BMAP_4_, *K* = 25	3.7622	3.9683	4.0532	5.3351
BMAP_5_, *K* = 25	5.4671	5.6264	5.6730	6.7445
BMAP_1_, *K* = 50	1.3455	1.5215	1.6623	4.5395
BMAP_2_, *K* = 50	1.7317	2.0467	2.1777	4.2515
BMAP_3_, *K* = 50	3.9533	4.1060	4.1880	5.9480
BMAP_4_, *K* = 50	4.3168	4.5536	4.6513	6.1946
BMAP_5_, *K* = 50	6.3348	6.5241	6.5816	8.0693
BMAP_1_, *K* = 100	1.3523	1.5317	1.6748	4.6437
BMAP_2_, *K* = 100	1.7752	2.1011	2.2370	4.4212
BMAP_3_, *K* = 100	4.0998	4.2638	4.3463	6.2562
BMAP_4_, *K* = 100	4.8728	5.1418	5.2528	7.0180
BMAP_5_, *K* = 100	6.8408	7.0518	7.1170	8.8153

The resulting burst ratios, obtained via Theorem 1 for buffer sizes of 25, 50 and 100, are shown in Table 1.

As we can see, the burst ratio grows with the complexity of the arrival stream. Both the autocorrelated arrivals and batch structure make *B* large, and even larger, when combined together. For instance, for *K* = 50 and the constant service time, the value of *B* grows almost 5 times from BMAP_1_ to BMAP_5_, and this change is caused only by the structure of the arrival stream—all the other system parameters are unaltered. This should constitute a clear warning: computing the burst ratio via a simple Poisson model makes no sense, if the real stream has a rich internal structure.

What is a little surprising in Table 1 is that *B* is greater in the cases with the alternating autocorrelation (BMAP_5_) than in the cases with the positive autocorrelation (BMAP_4_).

As we can also see in Table 1, the burst ratio grows with the coefficient of variation of the service time and this effect occurs for all considered arrival processes. Moreover, this effect is quite strong on its own, in the sense that for a high coefficient of variation, the burst ratio can be high even for a simple arrival proces, with no batches and no autocorrelation (see the results for the hyper-exponential distribution).

Finally, in Table 1 we can observe that the burst ratio grows with the buffer size, *K*. In the next subsection, we will study this effect in detail, depending of the internal structure of BMAP.

### 6.1 Dependence on the buffer size

To study the dependence of the burst ratio on the buffer size, we assume the load of 1, choose one distribution of the service time, i.e. constant equal to 1/6.666666, and vary *K* from 1 to 200.

**Fig 2:**
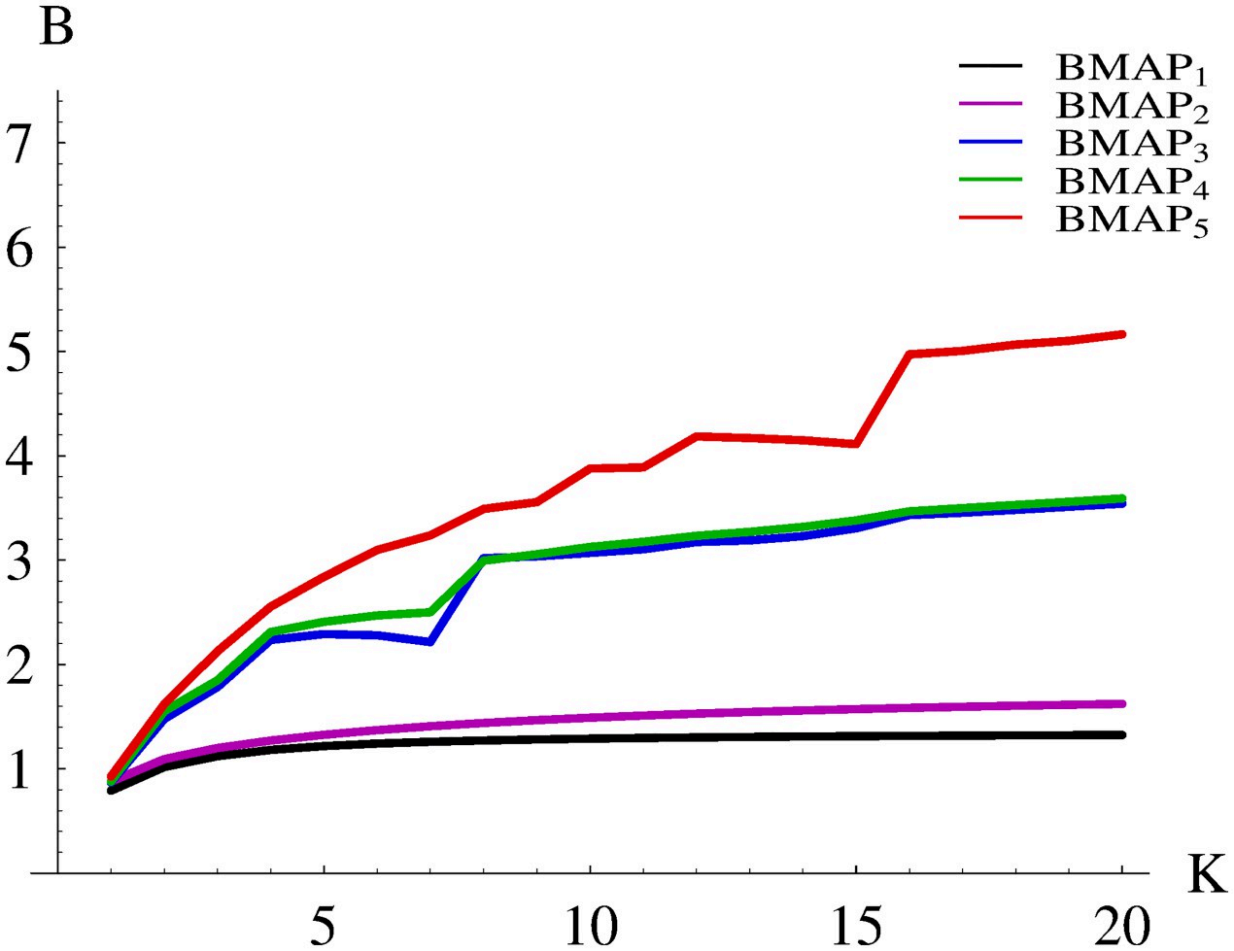
Dependence of the burst ratio on the buffer size for different BMAPs, range 1-20.

**Fig 3:**
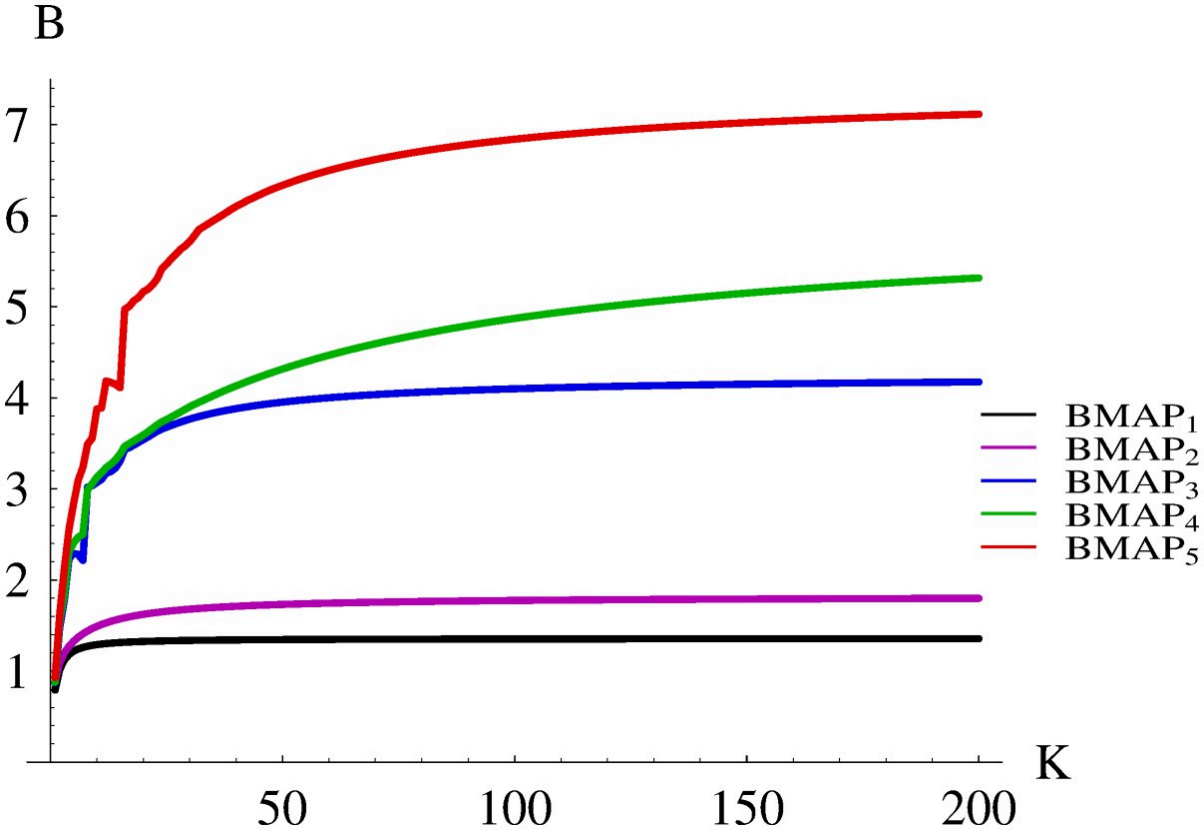
Dependence of the burst ratio on the buffer size for different BMAPs, range 1-200.

The resulting burst ratios for BMAP_1_-BMAP_5_ are shown in Figs 2 and 3. Namely, the range of buffer sizes 1-20 is depicted in Fig 2, while the full range 1-200, in Fig 3.

Firstly, it can be observed that the buffer size may influence the burst ratio profoundly. However, this effect depends on the complexity of the arrival stream—the more complex it is, the more variability for different buffers can be seen. For instance, in the case of BMAP_1_ the burst ratio grows by the factor of 1.7, when the buffer grows from 1 to 200. On the other hand, in the case of BMAP_5_ the burst ratio grows by the factor of 7.7, due to its complex internal structure.

As we can also see, for BMAP_1_ and BMAP_2_, which have no batch structure, the burst ratio grows monotonically with the buffer size. However, when batch arrivals are involved, this dependence may not be monotonic. For instance, in the case of BMAP_3_ we have *B* = 2.2923 for *K* = 5 and *B* = 2.2157 for *K* = 7. Similarly, in the case of BMAP_5_ we have *B* = 4.1858 for *K* = 12 and *B* = 4.1118 for *K* = 15. It is easy to see that such irregularities in Fig 2 happen for multiplications of the batch size, e.g. around buffer sizes of 8 and 16. They are connected with the possibility of accepting the whole arriving batch (or more than one batch) to the buffer.

Another interesting observation in Fig 2 is that for *K* = 1 the burst ratio is less than 1, for every considered arrival stream (it varies from 0.7909 for BMAP_1_ to 0.9276 in BMAP_5_). In most cases, the queueing mechanism makes *B* greater than 1 and situations with *B* < 1 are rare. As we can see, such result can be forced by using an extremely small buffer, at the cost of high loss ratio.

Finally, in Fig 3 we can see that the burst ratio approaches a limit as the buffer size grows. The limit is achieved rather quickly when there are no autocorrelation and no batch arrivals. The limit is approached more slowly when the autocorrelation and batch arrivals are involved. The slowest convergence can be observed for batch arrivals and positive autocorrelation.

### 6.2 Dependence on the system load

In this subsection we study the dependence of the burst ration on the system load. To accomplish this, we assume *K* = 50 and constant service time, which varies from 0.015 to 0.3. Therefore, the system load varies from a severe underload (*ρ* = 0.1) to a severe overload (*ρ* = 2).

**Fig 4:**
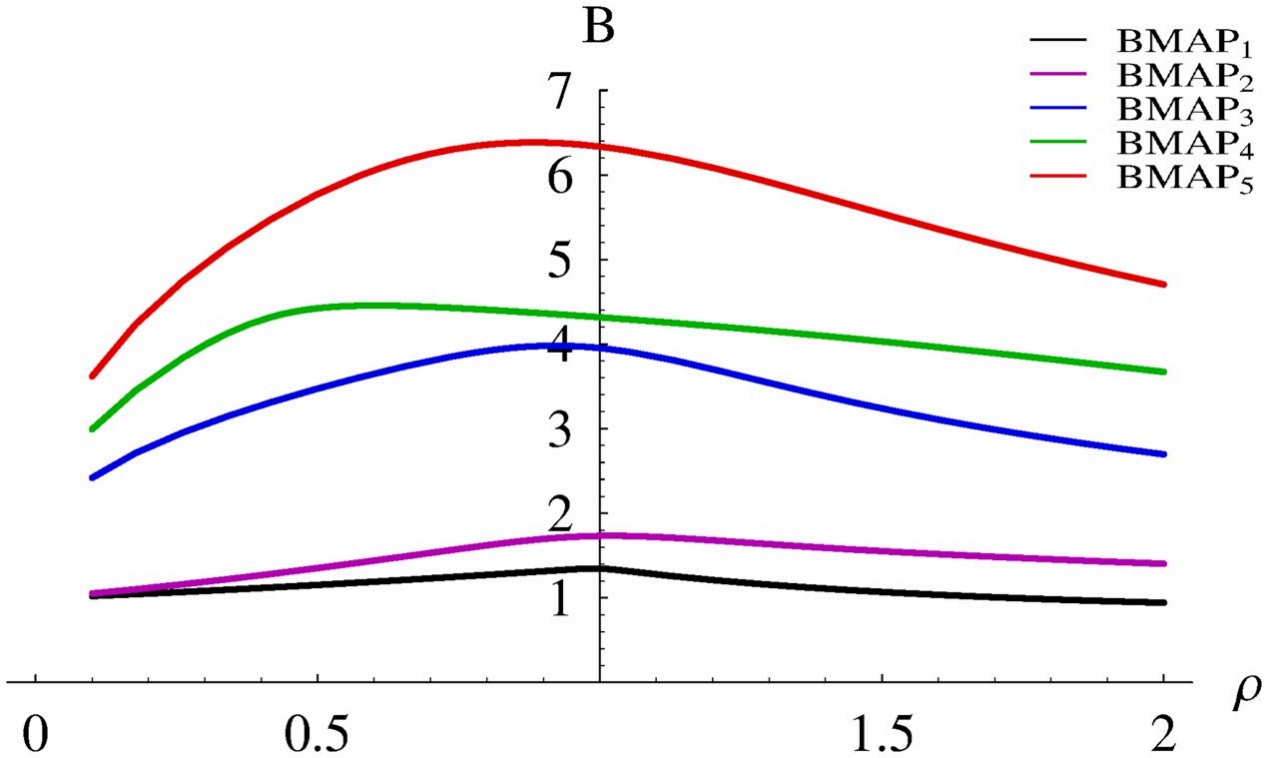
Dependence of the burst ratio on the system load for different BMAPs.

The resulting burst ratios for BMAP_1_-BMAP_5_ are depicted in Fig 4.

The first striking observation is that the load influences the burst ratio much less than the buffer size. For single arrivals in BMAP_1_ and BMAP_2_, the curves are almost completely flat. The more complex the arrival stream, the less flat the curve is, but generally the variability is much smaller than in Fig 4.

We can observe also that for every considered BMAP, the dependence is non-monotonic, with a maximum. The more simple the arrival process, the closer this maximum is to *ρ* = 1. Batch arrivals and/or autocorrelation cause shifting of this maximum to smaller values of *ρ*. For instance, in the case of BMAP_4_, this maximum occurs for as low *ρ* as 0.6.

Finally, it can be observed that for extremely high loads the burst ratio can get below 1. For instance, in the case of BMAP_1_ and *ρ* = 2 we have *B* = 0.9421.

### 6.3 Verification in simulations

Theorem 1 was also verified in a set of simulations. For this purpose, discrete-event simulator Omnet++, [31], was exploited. Note that simulation of the BMAP process is not demanding, if we use its constructive definition based on probabilities *p*_*i*_(*j*, *k*) given in the third paragraph of Section 3.

Several simulation experiments were carried out, with different BMAP parameterizations, loads, buffer sizes and service time distributions. In every simulation run, 100 milion of jobs were processed by the queueing system, while the empirical burst and loss ratios were computed. In every case, a high agreement between simulation results and Theorem 1 was obtained.

In Table 2, a few selected results for different system parameterizations are presented.

**Table 2:** Simulated and theoretical loss and burst ratios for different system parameterizations.

system parameters	simul. *L*	theor. *L*	simul. *B*	theor. *B*
BMAP_1_, *K* = 100, *ρ* = 1.5, const. service	0.3333	0.3333	1.0693	1.0693
BMAP_2_, *K* = 80, *ρ* = 1.2, uniform service	0.1673	0.1673	2.0097	2.0094
BMAP_3_, *K* = 60, *ρ* = 1.0, Erlang service	0.0623	0.0624	4.2361	4.2364
BMAP_4_, *K* = 40, *ρ* = 0.9, const. service	0.2737	0.2739	4.1800	4.1801
BMAP_5_, *K* = 20, *ρ* = 0.7, uniform service	0.2222	0.2224	5.3673	5.3683
BMAP_4_, *K* = 40, *ρ* = 1.0, hyper-exp. service	0.3186	0.3185	5.9161	5.9163
BMAP_5_, *K* = 60, *ρ* = 1.0, hyper-exp. service	0.1436	0.1436	8.3085	8.3083

## 7 Conclusions

We derived an explicite formula for the burst ratio in a finite-buffer queue fed by a versatile arrival process, BMAP, which among other things enables modeling of the interarrival time distribution, autocorrelation and batch arrivals. As it was discussed and demonstrated, the formula can be easily applied to obtain numbers via numerical calculations.

The study was motivated by networking, where both the BMAP process and the burst ratio parameter are of interest. However, due to the versatile nature of the arrival process, and the used general language of the queueing theory, the results can be applied in other fields with ease.

As was shown in numerical examples, several parameters of the system may influence the burst ratio. The picture is quite complicated due to the complex structure of the arrival process, which may or may not posses some important features (batches or autocorrelation), or posses them to a different extent (strength of the correlation, sizes of batches), or in a different way (alternating versus positive correlation).

For instance, we have seen that the burst ratio depends strongly on the buffer size. In some examples, extremely high *B* was obtained, above 7, while in others, *B* was below 1. In general, for large buffers the burst ratio achieves a limit, which depends deeply on the batch and/or autocorrelation structure. But for small or moderate buffers, dependence of *B* on *K* may not be monotonic—the curve may be distorted at multiplications of batch sizes. This is not observed in models without batch arrivals. We have seen also that the burst ratio depends on the system load in a non-monotonic way, and a maximum can be observed in the curve. For an arrival stream of a simple structure, this maximum is close to *ρ* = 1, what has been already observed in [18]. However, the more complex structure of the arrival stream, the more this maximum is shifted towards 0 (herein 0.6 in one of the examples).

These and other results discussed in Section 6 suggest, that when computing the burst ratio, we have to model precisely the structure of the arrival process, the service time and the buffer size. Modeling all of these features is allowed by the model studied herein.

Perhaps the most interesting future work on the burst ratio will be deriving it in models of active queue management (see, e.g. [32, 33] and the references given there). In active queue management, the arriving job or packet may be rejected even if the buffer is not full. The rejection is typically probabilistic, i.e. every arriving job can be rejected and the probability of rejection is a more or less complicated function of system characteristics, for instance the queue length. Sample derivation of the burst ratio in an active queue management model can be found in [34], but only for extremely simple arrival and service processes (both exponential). Active queue management is now in high demand, to mitigate the bufferbloat phenomenon in the Internet, [35]. One of the positive effects of its application may be a reduction of the burst ratio and, as a consequence, improvement of the quality of real-time multimedia transmissions.

Another interesting future work would be searching for formulas alternative to (11) and (15). In their current form, these formulas contain subtraction operation, which may cause numerical instability when the buffer size is large.
